# 3D-Printed Alginate/Pectin-Based Patches Loaded with Olive Leaf Extracts for Wound Healing Applications: Development, Characterization and In Vitro Evaluation of Biological Properties

**DOI:** 10.3390/pharmaceutics16010099

**Published:** 2024-01-11

**Authors:** Francesco Patitucci, Marisa Francesca Motta, Marco Dattilo, Rocco Malivindi, Adele Elisabetta Leonetti, Giuseppe Pezzi, Sabrina Prete, Olga Mileti, Domenico Gabriele, Ortensia Ilaria Parisi, Francesco Puoci

**Affiliations:** 1 Department of Pharmacy, Health and Nutritional Sciences, University of Calabria, 87036 Rende, CS, Italy; francesco.patitucci@unical.it (F.P.); marisafrancesca.motta@unical.it (M.F.M.); marco.dattilo@unical.it (M.D.); rocco.malivindi@unical.it (R.M.); adeleelisabetta.leonetti@unical.it (A.E.L.); gpezzi97@gmail.com (G.P.); sabrina.prete@unical.it (S.P.); francesco.puoci@unical.it (F.P.); 2 Macrofarm s.r.l., c/o Department of Pharmacy, Health and Nutritional Sciences, University of Calabria, 87036 Rende, CS, Italy; 3 Department of Information, Modeling, Electronics and System Engineering, University of Calabria, 87036 Rende, CS, Italy; o.mileti@dimes.unical.it (O.M.); domenico.gabriele@unical.it (D.G.)

**Keywords:** 3D printing, patches, wound healing, wound management, alginate, pectin, olive leaf extracts (OLEs), scratch assay, biocompatibility, human cell line activation test (h-CLAT)

## Abstract

Traditional wound dressings may lack suitability for diverse wound types and individual patient requirements. In this context, this study aimed to innovate wound care by developing a 3D-printed patch using alginate and pectin and incorporating Olive Leaf Extract (OLE) as an active ingredient. Different polymer-to-plasticizer ratios were systematically examined to formulate a printable ink with optimal viscosity. The resultant film, enriched with OLE, exhibited a substantial polyphenolic content of 13.15 ± 0.41 mg CAE/g, showcasing significant antioxidant and anti-inflammatory properties. Notably, the film demonstrated potent scavenging abilities against DPPH, ABTS, and NO radicals, with IC_50_ values of 0.66 ± 0.07, 0.47 ± 0.04, and 2.02 ± 0.14 mg/mL, respectively. In vitro release and diffusion studies were carried out and the release profiles revealed an almost complete release of polyphenols from the patch within 48 h. Additionally, the fabricated film exhibited the capacity to enhance cell motility and accelerate wound healing, evidenced by increased collagen I expression in BJ fibroblast cells. Structural assessments affirmed the ability of the patch to absorb exudates and maintain the optimal moisture balance, while biocompatibility studies underscored its suitability for biomedical applications. These compelling findings endorse the potential application of the developed film in advanced wound care, with the prospect of tailoring patches to individual patient needs.

## 1. Introduction

Skin is a barrier that protects the body from the aggression of external agents such as bacteria, fungi and other pathogens. At the moment of a skin injury, diverse intracellular and intercellular pathways are initiated to facilitate the restoration of tissue integrity and homeostasis. This process, called wound healing, refers to a restoration of damaged tissue by renewed tissue, entailing inflammation and blood clotting, tissue formation (cell proliferation) and tissue remodeling (maturation and cell differentiation) [1,2]. These phases are not consecutive but overlap with each other. In each phase of wound healing, different cell types are involved, such as macrophages, leucocytes, fibroblasts and keratinocytes, and their production is in turn regulated by several factors (cytokines, growth factors) [3].

Essentially, the skin consists of three layers of tissue: the epidermis, a keratinized multilayer; the dermis, a thick underlying layer of connective tissue; and the hypodermis, a subcutaneous layer of fat. After a wound event, a temporary repair is achieved via the coagulation of blood and, then, a cascade of regeneration processes is initiated both in the epidermis and dermis to form contractile granulation tissue that brings the wound edges together [4,5]. In healthy individuals, a simple wound can heal in a few days, whereas in chronic wounds, the healing process does not occur normally; it is interrupted and a chronic inflammation appears, prolonging the healing process. Today, chronic wounds represent a major clinical problem. In 2019, it is estimated that around 6 million people worldwide suffer chronic wounds and 85% of these subjects are elderly; these numbers result in a huge financial health care of up to USD 3 billion per year [6].

Numerous studies are focused on the development of new drugs that promote the healing of chronic wounds. In fact, the healing process can be slowed down by the presence of a concomitant disease (such as diabetes mellitus) and the result is poor healing, from which a chronic wound is formed [7].

In this context, recent studies have focused their attention on natural compounds due to their antioxidant and anti-inflammatory properties [8]. Phenolic compounds, specifically polyphenols, are a group of secondary metabolites of plants that exhibit potential in the therapeutic intervention for diverse cutaneous injuries, including burns and wounds [9]. The wound healing process is aided by the beneficial properties of polyphenols found in certain plant species including *Bacopa procumbens*, which has traditionally been used in wound healing due to the presence of phenolic compounds that are able to regulate cell proliferation, adhesion and differentiation, and improve cell migration [10]. In 2022, indeed, Martínez-Cuazitl et al. described the effects of polyphenols from *Bacopa procumbens* on the wound healing process and incorporated these active molecules into a hydrogel formulation, which was assessed in a rat excision wound model showing that the treatment accelerated wound closure and reduced inflammation, enhancing cell proliferation and collagen organization [11].

Polyphenols contained in olive leaves are another example demonstrating beneficial effects in wound healing. In particular, these compounds are known to increase the deposition of collagen fibers and promote the process of re-epithelization, increasing angiogenesis processes and modulating inflammatory cytokines [12]. The major bioactives contained in olive leaves are oleuropein, hydroxytyrosol, chlorogenic acid, caffeic acid, verbascoside and rutin. Among them, oleuropein is the main polyphenol contained in olive leaf extracts and it is widely used to treat skin diseases and wounds, showing the ability to accelerate the healing process [13,14,15].

In order to manage wounds, hydrogels are an excellent dressing for skin repair. The delivery of the polyphenols contained in olive leaves via hydrogels has more advantages than other forms of delivery in terms of size, stability and antioxidant properties. In fact, numerous studies report the use of hydrogels to stop bleeding, avoid bacterial infections and increase collagen formation [16,17]. In addition, hydrogels allow the retention of high levels of water and possess good swelling capacity, an ability that is useful in the absorption of exudate from the wound site [18]. In this context, alginate is well known for its high biocompatibility and gelling capacity, making it an essential component for the production of hydrogels. Alginate hydrogels are widely used in wound care due to the similarity of their structural properties to extracellular tissue matrices, maintaining a physiologically moist environment, reducing bacterial infection at the wound site and facilitating wound healing [19]. In addition, pectin shows non-toxicity, biocompatibility, mucoadhesiveness, water absorption capacity and anti-inflammatory properties, being able to produce a gel with enhanced properties [20]. Furthermore, it forms gels in the presence of Ca^2+^ ions, like alginate [21].

In order to keep up with current advancements, this research study aimed to use a lab-made 3D printer, as this technology is increasingly being used to produce biomedical devices. Specifically, 3D printing is intended for use as a more precise production process than conventional hydrogel preparation methods, and has a very short prototyping time [22]. For this purpose, Semi-Solid Extrusion (SSE) printing, also known in the literature as Pressure-Assisted Microsyringe (PAM), was employed and, since this technology requires a semi-solid formulation as a starting material, it has numerous advantages: it uses of a wide variety of polymers (not only thermoplastic polymers), formulations or doses can be customized to individual needs, precise drug encapsulation is achieved, and it is suitable for thermosensitive drugs [23]. In addition, high temperatures are not required and the stringent quality standards expected by regulatory organizations are guaranteed by the use of disposable syringes and pre-filled cartridges [24].

In agreement with the background, this research work was focused on the development of a 3D-printed patch for wound healing applications that was based on the bioactive compounds present in Olive Leaf Extracts (OLEs). The patches were developed using pectin and alginate as polymeric materials and glycerol as a plasticizer. The semi-Solid Extrusion technique was used as the 3D printing technology and the obtained films were characterized as follows.

## 2. Materials and Methods

### 2.1. Materials

Sodium alginate (ALG, Prod. Num. 180947), pectin (PEC, Prod. Num. P9135), glycerol (GLY, Prod. Num. G9012), calcium chloride (Prod. Num. 1.02378), Folin-Ciocalteu reagent (2 N, Prod. Num. F9252), sodium carbonate (Prod. Num. 223530), catechin (CA, Prod. Num. 22110), 2,2-diphenyl-1-picrylhydrazyl radical (DPPH, Prod. Num. D9132), 2,2′-azinobis-(3-ethylbenzothiazoline-6-sulfonic acid) (ABTS, Prod. Num. A1888), potassium persulfate (KPS, Prod. Num. 216224), sodium nitroprusside (SNP, Prod. Num. 13451), Griess reagent (Prod. Num. 03553), sulfuric acid (Prod. Num. 258105), sodium phosphate (Prod. Num. 342483), ammonium molybdate (Prod. Num. 09878), disodium hydrogen phosphate (Prod. Num. 71640), sodium dihydrogen phosphate (Prod. Num. 71496), gelatin (Prod. Num. 48723), Eagle’s Minimum Essential Medium (MEM, Prod. Num. M0446), Dulbecco’s Modified Eagle Medium (DMEM, Prod. Num. D0822), Penicillin/Streptomycin (Prod. Num. P0781), β-Mercapto-ethanol (Prod. Num. M3148), Fetal Bovine Serum (FBS, Prod. Num. F4135), Bovine Calf Serum (BCS, Prod. Num. 12133C), 3-(4,5-dimethylthiazol-2-yl)-2,5-diphenyltetrazolium (MTT, Prod. Num. 475989), paraformaldehyde (Prod. Num. P6148), Triton X-100 (Prod. Num. T8787), bovine serum albumin (Prod. Num. A2153), 4′,6-diamidino-2-phenylindole (DAPI, Prod. Num. D8417), phycocyanin (Prod. Num. P2172), Neutral Red (NR, Prod. Num. N4638), acetic acid (Prod. Num. 695092), nickel sulphate (Prod. Num. 1.06726), propidium iodide (Prod. Num. 537059), FACS buffer (PBS, 0.5–1% BSA or 5–10% FBS, 0.1% NaN_3_ sodium azide), sodium azide (Prod. Num. 71289), Bovine Serum Albumine (BSA, Prod. Num. A4503) and γ globulin (Prod. Num. G5009) were purchased from Sigma-Aldrich s.r.l. (Milan, Italy).

RPMI 1640 medium was obtained from ATCC (Manassas, VA, USA).

Collagen I Polyclonal Antibody and goat pAb IgG (TRITC) were purchased from INVITROGEN (Carlsbad, CA, USA) and Abcam (Cambridge, UK), respectively.

A high-capacity cDNA reverse transcription kit and stealth RNAi negative ctl kit were obtained from Life Technologies (Carlsbad, CA, USA).

iTaq™ Universal SYBR^®^ Green, Microseal ‘B’ PCR Plate Sealing Film and Hard-Shell^®^ 96-Well PCR Plates were purchased from Bio-Rad (Hercules, CA, USA).

All solvents were reagent or HPLC grade and obtained from VWR (Milan, Italy).

The olive leaves powder was supplied by Biocal s.r.l. (Luzzi, CS, Italy).

### 2.2. Cell Lines and Culture Conditions

The BJ (human fibroblast), Balb/3T3 Clone A31 (murine fibroblast) and THP-1 (human monocyte) cell lines were obtained from the American Type Culture Collection (ATCC) located in Manassas, VA, USA.

The BJ, Balb/c 3T3 and THP-1 cells were cultured in MEM containing 10% FBS and 1% Penicillin/Streptomycin, DMEM containing 10% BCS and 1% Penicillin/Streptomycin, and RPMI 1640 containing 10% FBS, 1% Penicillin/Streptomycin and 0.05% β-Mercapto-ethanol, respectively.

All cell lines were maintained at a temperature of 37 °C in a modified atmosphere composed of 5% humidified CO_2_.

### 2.3. Preparation of the Olive Leaf Extract (OLE) Powder

The Olive Leaf Extract (OLE) powder was prepared starting from a powder of olive leaves powder, which was supplied by Biocal s.r.l. (Luzzi, CS, Italy), and the adopted extraction procedure was a slightly modified version of a protocol reported in the literature [25].

In detail, olive leaves from *Olea europaea* L. (Carolea cultivar) were harvested from the region of Calabria, Italy, and cold dried at low temperatures (≤20 °C) with an air flow rate of 5 m/s to obtain a minimally processed dried product without the loss of its organoleptic and quality properties. Then, the dried leaves were ground into a fine powder, which was mixed with distilled water at a drug/extraction solvent ratio of 3:10 (*w*/*w*). The obtained mixture was magnetically stirred at 40 °C and, after 2 h, centrifuged at 8000 rpm for 10 min. The supernatant was filtered using Whatman no. 4 filter paper and the extraction process was repeated on the pellet two more times.

The extracted fractions were combined and, finally, freeze-dried using a Freeze Dryer Micro Modulyo (Edwards Ltd., Surrey, UK).

### 2.4. Preparation of the 3D-Printable INKs

The 3D-printable INKs were prepared by dissolving different amounts of sodium alginate, pectin and glycerol, which was used as a plasticizer, in 100 mL of an OLE aqueous solution (1%, *w*/*v*). The mixing was performed via mechanical stirring under vacuum in order to remove bubbles.

Different amounts of polymers and plasticizer were tested, while control 3D-printable INKs, with the same composition but using distilled water instead of the OLE aqueous solution, were also prepared.

Preliminary viscosity studies were carried out in order to identify the optimal INK composition, which had to be visually homogenous, easily extrudable and, at the same time, suitable to obtain a uniform 3D-printed polymeric film [26]. The viscosity measurements were performed with a PCE-RVI 4 rotational viscometer (PCE Instruments UK Ltd., Southampton, UK), an instrument capable of determining the viscosity according to the Brookfield Method.

### 2.5. Fabrication of the 3D-Printed Patches

The 3D-printed patches containing OLE were prepared with a lab-made 3D printing system employing a commercial extrusion-based printer that had been modified with a syringe-based extrusion mechanism for the Semi-Solid Extrusion (SSE) 3D printing method.

Using FreeCAD 0.20.2 as the 3D modeling software, a 3D-printed model with dimensions of 20.0 mm in X, 20.0 mm in Y, and 0.2 mm in Z was prepared.

The designed file was imported into Repetier Host software V2.2.4 as a stereolithography (.stl) file to slice, preview, and print films. The following printer settings were established: Nozzle size (1 mm), layer height (0.1 mm), print speed (70 mm/s), travel speed (150 mm/s), and printing temperature (25 °C).

After printing, the obtained films containing the olive leaf extract (OLEFs—Olive Leaf Extract Films) were dried for 24 h at 40 °C on the 3D printer plate.

During the next step, the samples were poured into glass vessels and crosslinked via immersion in a CaCl_2_ aqueous solution. In detail, a series of crosslinking solutions of different concentrations (90.11, 135.16 and 180.21 mM CaCl_2_) were prepared. Then, the 3D-printed films were immersed into these solutions for 20, 40 and 60 sec and, thus, by varying the immersion time. Finally, the samples (Table S1) were rinsed with distilled water to remove the excess of CaCl_2_ and dried overnight at 37 ± 0.5 °C.

In order to test the reproducibility of the 3D prints and to give the most accurate results, the thickness was measured at five distinct sites, specifically in the middle and at the corners, by a laboratory micrometer.

Blank patches, acting as control samples, were also printed according to the reported procedure and using 3D-printable INKs that were prepared dissolving alginate, pectin and glycerol in distilled water.

### 2.6. Differential Scanning Calorimetry (DSC) and Scanning Electron Microscope (SEM) Analyses

An amount of 10 mg of the analyzed sample (3D-printed patch, 3D-printed blank patch and OLE powder) was weighed and placed inside a hermetically sealed aluminum pan, and the thermal degradation behaviors were determined using a DSC200 PC differential scanning calorimeter (Netzsch, Selb, Germany) performing the analyses from 25 °C to 500 °C under a heating rate of 10 °C·min^−1^.

The surface morphology of the studied items was examined using a Scanning Electron Microscope (SEM) (FlexSem 1000 III, Hitachi, Tokyo, Japan). In order to facilitate the analysis, small pieces of the films were prepared and affixed to the SEM metallic stub using double-sided adhesive tape. The analysis was performed at 10 kV and micrographs were acquired at varying degrees of magnification.

### 2.7. Film Expansion Profile

In order to test the fluid uptake or exudate absorption in the wound environment, a film expansion model was assessed [27].

A 4% (*w*/*v*) gelatin aqueous solution was prepared under continuous stirring at a temperature of 45 °C; after 1 h, the solution was kept at room temperature and allowed to solidify overnight. Film samples with an area of 3 cm^2^ (1.5 cm × 2.0 cm) with or without extract were placed on the surface of the gelled gelatin solution and positioned in a thermostatic bath at 37 °C for 72 h, evaluating the expansion profile at predetermined time intervals (1, 2, 4, 6, 8, 24 and 48 h) using Equation (1):

(1) $$Expantion\left(\%\right)=\frac{D_{t}-D_{0}}{D_{0}}\times 100$$

where D_t_ and D_0_ were the diameters of the film after and before expansion, respectively.

The experiments were performed in triplicate.

### 2.8. Water Vapor Transmission Rate (WVTR)

The Water Vapor Transmission Rate (WVTR) was evaluated following a protocol reported in the literature [28].

The patch samples were cut into circular shapes (18.5 mm), fixed with parafilm to the entrance of the tubes filled with distilled water (10 mL) and incubated at 37 °C at a relative humidity of 85%. An open test tube containing only deionized water was used as a control. After 48 h, the test tubes were weighed and the water vapor permeability of the samples was determined using Equation (2):

(2) $$WVTR\left(\frac{g}{m^{2}\cdot d}\right)=\frac{W_{t}-W_{0}}{A\cdot t}\times 100$$

where W_0_ denotes the initial weight of the item under examination, W_t_ represents the weight at the considered time interval, t is the time in days and A is the area of the studied item.

The experiments were performed in triplicate.

### 2.9. Total Phenolic Content (TPC)

The polyphenol content was determined using the Folin–Ciocalteu reagent procedure, following the protocol reported in the literature with some modifications [29].

A mixture containing 1 mL of Folin–Ciocalteu reagent, 1 mL of a 7.5% (*w*/*v*) Na_2_CO_3_ solution and 1 mL of distilled water was added to 50 mg of each patch and incubated for 2 h under agitation at room temperature. Then, the solution was filtered and the absorbance at 760 nm was measured, employing an Evolution 201 UV/Vis spectrometer (Thermo Fisher Scientific, Waltham, MA, USA), against a control in the absence of the tested items.

The adopted experimental procedure was also employed to evaluate the impact of the polymeric materials, such as alginate and pectin, on the performed assay when applied to the control patches.

In order to quantify the amount of total phenolic compounds, the calibration curve of catechin (CA) was used to calculate the catechin equivalents (CAE) in milligrams per g of sample (mg CAE/g).

The assay was performed in triplicate.

### 2.10. Evaluation of the Antioxidant Properties

#### 2.10.1. Total Antioxidant Activity

The total antioxidant activity of the 3D-printed films was evaluated according to the literature [30].

For this purpose, 5 mg of each sample was reacted with 2.4 mL of the previously prepared reagent solution and 0.6 mL of distilled water; this was then incubated at 95 °C. After 150 min, the samples were cooled and filtered to determine the absorbance at 695 nm against a control prepared in the absence of the studied films.

The adopted experimental procedure was also employed to evaluate the impact of the polymeric materials on the performed assay when applied to the blank patches.

The total antioxidant activity was calculated in terms of the catechin milligrams equivalents per gram of film (mg CAE/g), according to the CA calibration curve.

The experiments were carried out in triplicate.

#### 2.10.2. DPPH Assay

The scavenger activity against 2,2′-diphenyl-1-picrylhydrazyl (DPPH) and 2,2′-azino-bis(3-ethylbenzothiazoline-6-sulfonic acid) (ABTS) radicals was investigated in accordance with the relevant literature [31].

For the DPPH radical scavenging assay, an ethanolic solution of the radical (0.19 mM) was prepared. Known samples weights (5, 7, 10, 12 and 15 mg) were reacted with 6 mL of the DPPH reagent and 4 mL of distilled water. After 15 min, the samples were filtered and the absorbance at 517 nm was evaluated against a control prepared in the absence of the items under investigation.

The adopted experimental procedure was also employed to evaluate the impact of the polymeric materials, such as alginate and pectin, on the performed assay when applied to the control patches.

The antioxidant activity was expressed as the percentage of DPPH radical inhibition (Equation (3)):

(3) $$Inhibition\left(\%\right)=\frac{A_{0}-A_{1}}{A_{0}}\times 100$$

where A_0_ is the absorbance of the control and A_1_ is the absorbance of the sample.

The experiments were performed in triplicate.

#### 2.10.3. ABTS Assay

ABTS was dissolved in distilled water to obtain a solution with a concentration of 7 mM. The radical was produced as a result of the reaction between ABTS and potassium persulfate (final concentration 2.45 mM) and by leaving the mixture to stand protected from light at room temperature overnight. The resulting blue-green solution was diluted at a ratio of 1:35 with distilled water to obtain the 0.194 mM reagent solution. Known weights of the samples (5, 7, 10, 12 and 15 mg) were reacted in the dark under agitation with 16 mL of the reagent and 4 mL of distilled water. After 5 min, the samples were filtered and the absorbance was measured at 734 nm.

The adopted experimental procedure was also employed to evaluate the impact of the polymeric materials, such as alginate and pectin, on the performed assay when applied to the control patches.

The antioxidant activity was expressed as the percentage of the ABTS radical inhibition according to Equation (3).

The ABTS assay was carried out in triplicate.

### 2.11. Evaluation of the Anti-Inflammatory Properties: Nitric Oxide Scavenging Activity

The ability of the 3D-printed patches to scavenge nitric oxide radicals was assessed according to the literature, with slight modifications [32].

An aliquot of the sample (5, 7, 10, 12 and 15 mg) and 5 mL of a 5 mM sodium nitroprusside solution in PBS (pH 7.4, 10^−3^ M) were combined, and the reaction was conducted for 3 h at 25 °C under an UV lamp (25 W tungsten lump). Then, the resulting solution was filtered and 0.5 mL of the sample was reacted with 0.5 mL of Griess reagent, followed by the detection of the absorbance at 546 nm against a control prepared with the same procedure but without sample.

The adopted experimental procedure was also employed to evaluate the impact of the polymeric materials, such as alginate and pectin, on the performed assay when applied to the control patches.

The data were expressed as the percentage of nitric oxide inhibition according to Equation (3).

The experiments were carried out in triplicate.

### 2.12. In Vitro Release Studies

In vitro investigations on the release of polyphenols were conducted by employing the dialysis membrane technique [33].

For this purpose, 50 mg of the tested patch was inserted into a dialysis bag (Spectrum™ Labs Spectra/Por™ 5 12–14 kD MWCO) containing PBS at pH 7.4 (5 mL, 10^−3^ M). Then, the dialysis membrane was immersed in a glass vessel filled with 40 mL of PBS and placed on a thermostatically controlled magnetic stirrer (100 rpm) set at 37 ± 2 °C. Samples (2 mL) were collected at specific time points and changed with the same volume of fresh buffer to maintain the sink condition. Finally, the collected samples were analyzed using the Folin–Ciocalteu assay, as previously described.

The adopted experimental procedure was also employed to evaluate the impact of the polymeric materials on the performed studies when applied to the blank patches.

The in vitro release studies were conducted in triplicate.

### 2.13. In Vitro Diffusion Studies

In order to mimic skin permeability, an in vitro diffusion assay was carried out using Franz diffusion cells [34].

The donor chamber (upper) and the receptor chamber (bottom) were separated by Strat-M^®^ membranes (25 mm discs, Cat. No. SKBM02560, Merck Millipore, Waltham, MA, USA) that were cut into an area of 0.5 cm^2^, and the available diffusion area was 0.4614 cm^2^. Then, the 3D-printed patches (~20 mg) were placed onto the synthetic membrane. Each receptor chamber received 5 mL of 10^−3^ M PBS at pH 7.4, while 0.5 mL of the same buffer was inserted in each donor chamber. The apparatus was kept at 37 °C, and the receptor compartment was stirred at 600 rpm. Aliquots of 5 mL were removed at designated time points from the receptor chamber and replaced with fresh buffer. The amount of released polyphenols was quantified using the Folin–Ciocalteu assay, and the procedure employed for the quantification of polyphenols was the same as that used in Section 2.9.

The adopted experimental procedure was also employed to evaluate the impact of the polymeric materials on the performed studies when applied to the blank patches.

The in vitro diffusion studies were carried out in triplicate.

### 2.14. Cell Viability Assay

The cell viability was assessed using the 3-(4,5-dimethylthiazol-2-yl)-2,5-diphenyltetrazolium (MTT) assay [14].

The extraction of OLEF_9 and BF_9, which are, respectively, the 3D-printed patch loaded with OLE powder and the control patch prepared with the same composition of OLEF_9 but in the absence of the extract, was first performed according to ISO 10993-12:2021 [35].

In detail, the tested patches (1 mg/mL) were immersed in complete DMEM and incubated at 37 °C. After 24 h, the samples were filtered by means of filters with a porosity of 0.22 μm and the obtained patch extracts were diluted to 100 μg/mL.

BJ fibroblast cells (4 × 10^4^) were plated in 48 wells in complete medium and maintained in culture for 24 h. After incubation, the cells were starved in serum-free medium (SFM) for 12 h. Thereafter, treatments were performed with the BF_9 and OLEF_9 extracts at a concentration of 100 µg/mL. The test was performed at 3 different times: 24, 48 and 72 h. After each experimental time, 200 µL of MTT (0.3 mg/mL) was added for 3 h at 37 °C. After 3 h, the MTT solution was aspirated and 200 µL of DMSO was added to each well. The optical density at a wavelength of 570 nm was quantified by employing a Beckman Coulter microplate reader.

Eight replicates were performed for each sample.

### 2.15. Wound-Healing Scratch Assay

BJ fibroblast cells were grown to confluence in normal medium and then maintained in SFM for 12 h. Using a 200-microliter tip, scratches were formed on the monolayers to simulate a wound in the fibroblasts [14]. The cells were then treated with the BF_9 (50 and 100 µg/mL) or OLEF_9 (50 and 100 µg/mL) extracts. After 24 h, the healing process was documented by employing phase contrast microscopy at 4× magnification (CKX-53 Olympus).

The percentage of wound closure was calculated using the Image J 1.54 d software and expressed as reported in Equation (4):

(4) $$Wound\;Closure\;\left(\%\right)=\left[\left(A_{t}-A_{0}\right)/A_{t}\right]\times 100$$

where A_0_ is the area of the wound measured immediately after scratching and A_t_ is the area of the wound measured after 24 h.

### 2.16. Real-Time Reverse Transcriptase PCR Assay

Real-time RT-PCR was used to assess the gene expression in accordance with the specified methodology reported in the literature [36] and by employing SYBR Green Universal PCR Master Mix (Bio-Rad, Hercules, CA, USA).

The obtained cDNAs were amplified via PCR by using the following primers:
MMP-9 rev5′-CCTGCCAGTTTCCATTCATCMMP-9 fw5′-GCCATTCACGTCGTCCTTATGAPDH FW5′-CCCACTCCTCCACCTTTGAC-3GAPDH REV5′-TGTTGCTGTAGCCAA ATTCGT T-3′

The relative gene expression levels were normalized to a calibrator that was chosen to be the basal, vehicle-treated sample. The final results were expressed as the n-fold differences in gene expression relative to the GAPDH rRNA and calibrator, calculated using the ∆∆C_t_ method as follows: *n*-fold = 2^−(ΔCtsample − ΔCtcalibrator)^, where ∆Ct values of the sample and calibrator were determined by subtracting the average Ct value of the GAPDH rRNA reference gene from the average Ct value of the different genes analyzed.

### 2.17. Immunofluorescence

Firstly, 1.5 × 10^4^ cells were plated on immunofluorescence slides (ChamberSlides Thermo Scientific Nunc) and kept in culture for 24 h. Then, the cells were treated with the OLEF_9 and BF_9 extracts at a final concentration of 100 µg/mL for 24 h. After incubation, PBS washings were carried out and then 4% paraformaldehyde fixing in PBS for 20 min at room temperature was performed. Subsequently, the cells were permeabilized with Triton X-100 at 0.2% in PBS for 5 min and non-specific sites were blocked with an incubation with 5% bovine serum albumin for 30 min; this was followed by an incubation with anti-type 1 human collagen antibody (1:100) in PBS for one night at 4 °C.

On the subsequent day, the cells underwent triple washes with PBS followed by a 1 h incubation with secondary anti-mouse Goat pAb to Rb IgG (TRITC) (1:200) at room temperature. The negative control was incubated with normal mouse serum in place of primary antibody.

Finally, slides were mounted with 4′,6-diamidino-2-phenylindole (DAPI), which is commonly used as a nuclear counterstain in fluorescence microscopy and binds to nuclei, giving them a blue coloring. The collagen was visualized via marking with phycocyanin, which absorbs red-orange light in the electromagnetic spectrum at a wavelength of 620 nm and emits fluorescence at about 650 nm. Immunofluorescence analysis was performed under the OLYMPUS FV3000 microscope at 40× magnification.

### 2.18. Safety Assessment

#### 2.18.1. Neutral Red Uptake (NRU) Assay

The NRU test (ISO 10993-5:2009 “Biological evaluation of medical Devices-Part 5: Tests for in vitro cytotoxicity” [37]) was performed on Balb/3T3 Clone A31 cells [38].

The extraction of OLEF_9 and BF_9 according to ISO 10993-12:2021 was first performed as reported in Section 2.14 [35].

Then, 2.5 × 10^4^ cells per well were treated with the OLEF_9 and BF_9 extracts in DMEM at three final concentrations (25, 50 and 100 µg/mL) for 24 h at 37 °C and 5% CO_2_ atmosphere. After the treatment, cells were incubated with a 50 µg/mL NR solution for 3 h. After this time, the neutral red solution was aspired and the extraction procedure was conducted using a solvent mixture consisting of ethanol, water and acetic acid at a volumetric ratio of 50:49:1.

Absorbance at 540 nm was quantified using an Epoch microplate reader (BioTek, Winooski, VT, USA) and the viability (%) was determined according to Equation (5):

(5) $$\%Viability=\frac{{Abs}_{sample}-{Abs}_{blank}}{{Abs}_{control}-{Abs}_{blank}}$$

#### 2.18.2. Human Cell Line Activation Test (h-CLAT)

The h-CLAT (human cell line activation test) is a test that is performed to assess whether substances or mixtures have the potential to sensitive skin through the activation of the immune system. The test is performed according to the Organization for Economic Co-operation and Development (OECD) 442E and EURL 158-ECVAM (European Union Reference Laboratory for Alternatives to Animal Testing) protocol [38].

The experimental model for the test is represented by the THP-1 human leukemia monocytic cell line. To assess the activation of the immune system, the modulation of the expression of two costimulatory molecules, CD54 and CD86, is evaluated using NiSO_4_ as a positive control. THP-1 cells were grown in RPMI 1640 medium with 10% FBS, 1% Penicillin/Streptomycin and 0.05% mM β-Mercapto-ethanol.

In order to determinate the CV75 (concentration causing 25% mortality) to be used later, as reported in Test No. 442E: In Vitro Skin Sensitization, 1.5 × 10^5^ of cells per well were plated in a 96-well multi-well and incubated as described above. After incubation, the cells were treated with the samples at 8 serial concentrations. The next day, the medium with the treatment was removed via centrifugation, the cells were resuspended in FACS buffer containing PI and the CV75 was calculated via flow cytometry.

For the h-CLAT, 5 × 10^5^ cells per well were plated in a 24-well multiwell and incubated for 24 h. After incubation, treatments were added and the cells were incubated for 24 h. A 100 μg/mL nickel sulfate solution and culture medium were employed as the positive and negative control, respectively. After incubation, the cells were centrifuged, resuspended in FACS buffer and aliquoted into three parts. Next, they were centrifuged and resuspended in blocking solution (FACS buffer containing 0.01% γ globulin) for 15 min at 4 °C. Finally, the cells were incubated with a fluorescein antibody targeted to CD86, CD54 or IgG1, the latter being used as a control, for 30 min at 4 °C. After incubation, the antibody was removed via centrifugation, and two washes in FACS buffer were performed to remove excess antibody. Subsequently, the cells were resuspended with FACS containing PI. The CD54 and CD86 expression levels, as well as the cellular viability, were evaluated through flow cytometry analysis.

In order to assess whether the test chemical is to be considered a sensitizer, the Effective Concentration (EC) for CD86 and CD54 was determined. The EC, indeed, represents the concentration at which the studied item produced a RFI equal to 150 or 200, respectively.

The experiment was repeated in triplicate and on 3 different days. The EC150 and EC200 values are the median values calculated from three independent runs. When only two of three independent runs meet the positive criteria, the higher EC150 or EC200 value is adopted.

An increase in the CD54 and CD86 expression on THP-1 cells is correlated with the activation of an immune response due to exposure to a partially allergenic antigen.

#### 2.18.3. Hemolysis Assay

The hemolysis assay was carried out according to the procedure reported in the literature and by using the BF_9 (25 and 100 µg/mL) and OLEF_9 (25 and 100 µg/mL) extracts [39].

The study procedure with human blood was approved by the Ethics Committee of the University of Calabria (Unical AOO1 Central Administration, Doc. no. 234 of 14 January 2021).

### 2.19. Immunoblotting

The BJ1 cell lines were lysed in 500 μL of lysis buffer containing 50 mM of Tris-HCl at pH 8, 150 mM of NaCl, 1% Nonidet P-40, 0.5% sodium deoxycholate, 2 mM of sodium fluoride, 2 mM of EDTA, 0.1% sodium dodecyl sulfate, and a mixture of protease inhibitors (aprotinin, phenylmethylsulfonyl fluoride, and sodium orthovanadate) for efficient protein extraction.

Immunoblotting was carried out using the following antibodies: MMP-9 (MA5-32705 Rabbit Monoclonal—Thermo Fisher, Milan, Italy) and GAPDH (sc-32233, Santa Cruz Biotechnology, Milan, Italy).

The protein band intensities for the biological replicates were measured using the Scion Image laser densitometry scanning program (Scion Corporation, Frederick, MD, USA). A representative image from three independent experiments is presented.

### 2.20. Statistical Analysis

In vitro data were analyzed using Student’s *t*-test and by employing GraphPad Prism 8.3.0 (GraphPadSoftware, Inc., San Diego, CA, USA).

*p* < 0.05 was considered statistically significant.

## 3. Results and Discussion

### 3.1. Preliminary Characterization of INKs and 3D-Printed Films

Initially, the INKs composition was optimized and several studies were carried out for this purpose (only some of the tested INK formulations are shown in Table 1).

A thorough evaluation was made regarding the amount of OLE powder to be used in the INK production, since the components of the extract can influence the gel structure. OLE powder was chosen as the active ingredient due to its wound-healing properties, which were confirmed in the following characterization.

As a next step, the viscosity was measured to assess the printability of the INKs. In order to develop an optimal INK formulation suitable for printing, several polymer concentrations were tested and their viscosity was assessed. The optimal viscosity was obtained by evaluating the operating conditions and the printing hardware. An INK viscosity that is too high or too low, indeed, leads to clogging and, therefore, to the interruption or destructuring of the printout [40]. As shown in Table 1, the different INKs compositions led to a variation in the viscosity. Therefore, the final choices were INK_1 and INK_2, which had viscosity values between 4.0 and 8.0 Pa·s; this would enable the manufacture of compact, flexible and mechanically stable printouts. Additionally, the viscosity of INKs prepared in the absence of OLE powder, considered as blanks, was evaluated and the results were 2.5 and 3.6 Pa·s for blank INK_1 and blank INK_2, respectively. These data confirmed that the OLE powder imparted a higher viscosity to the gel, resulting in better structuring and firmness.

Based on these findings, it was decided that the studies on INK_2, which had an optimal viscosity, would be continued and several printing tests were performed.

**Figure 1:**
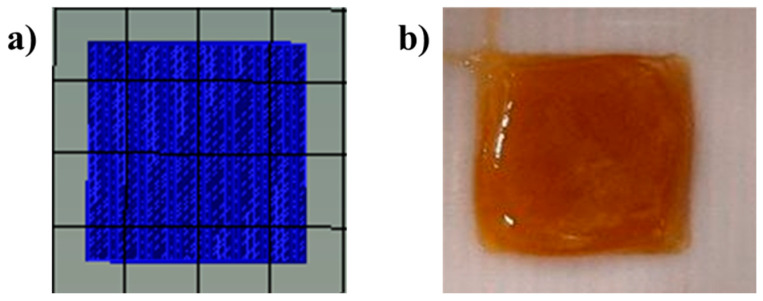
The 3D printer setting: (**a**) designed model; (**b**) 3D-printed film (not dried).

As shown in Figure 1, the printer settings produced films with a homogenous structure and the same dimensions as the used 3D printing model.

Regarding the 3D prints, the visual analysis of the films revealed no major changes between the produced samples, which were smooth and uniform in shape, with an average thickness of 0.220 ± 0.012 mm. The film thickness had to be measured because it is directly related to the reproducibility of 3D prints.

In order to increase the stability and, thus, avoid the rapid degradation of the samples, three different concentrations of the aqueous crosslinking solution and three different immersion times were examined by dipping the 3D-printed films obtained; this was starting from INK_2, as reported in Table S1. Alginates and pectins, indeed, are natural polysaccharides that find several applications in pharmaceutical and biomedical fields due to their ability to form insoluble hydrogels in the presence of divalent cations, such as calcium ions, which induce chain–chain associations consisting of chemical interactions between Ca^2+^ and guluronate and galacturonate residues in alginate and pectin, respectively. The different chemical compositions and molecular structures of the two polymeric materials lead to different crosslinking with Ca^2+^ ions. The egg-box model describes the mechanism of the Ca^2+^-dependent gelation of both alginate and pectin, which is based on the development of linkages between the carboxylate groups of polymers and the calcium ions [41,42].

Preliminary analyses were carried out and OLEF_7, OLEF_8, and OLEF_9 exhibited a good degree of crosslinking. These samples, indeed, visually retained their film structure and plasticity. In particular, OLEF_9 showed the best resistance, in terms of the maintenance of a coherent structure, even after 24 h of immersion in water. Therefore, this last one was chosen to proceed with the studies and a control patch was prepared with the same composition of OLEF_9 but in the absence of OLE powder (BF_9, Blank Film 9).

### 3.2. DSC and SEM Analyses

**Figure 2:**
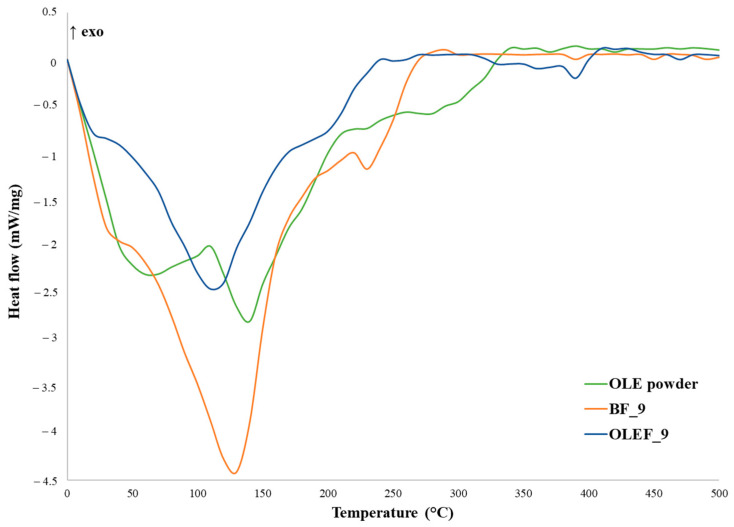
Structural characterization of the 3D-printed alginate/pectin-based patches: DSC thermographs obtained for OLE powder, BF_9 and OLEF_9.

Figure 2 illustrates the DSC curves of the OLE powder, BF_9 and OLEF_9.

The DSC analysis of the OLE powder displayed two endothermic peaks at 60 °C and 140 °C. The first one is quite broad and due to the release of free water; the second one is ascribable to the thermal degradation of the large variety of chemicals included in the olive leaf extract [43,44]. Oleuropein and hydroxytyrosol, indeed, are the major bioactives contained in olive leaves, and have melting points of around 65 °C and 55 °C, respectively [45,46].

The thermogram of BF_9 revealed a wide and intense endothermic peak around 130 °C that was attributed to the elimination of water molecules interacting with the hydroxyl and carboxylic acid groups of the polymers contained in the patch formulation [47].

On the other hand, OLEF_9 showed a less intense endothermic peak around 110 °C, indicating that water was lost from the polymer functional groups. However, the presence of the extract seems to contribute to the reinforcement of the film matrix.

**Figure 3:**
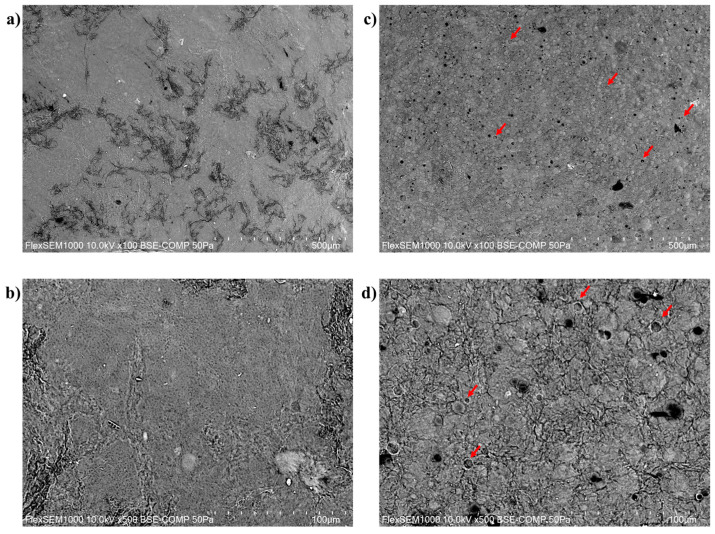
SEM images of the 3D-printed alginate/pectin-based patches: (**a**) BF_9 at 100× magnification; (**b**) BF_9 at 500× magnification; (**c**) OLEF_9 at 100× magnification; (**d**) OLEF_9 at 500× magnification. The red arrows indicate the pores.

The surface morphology of OLEF_9 and the corresponding blank film BF_9 was studied via Scanning Electron Microscopy (SEM) and the images of both 3D-printed films were captured at two different magnifications (100× and 500×), as shown in Figure 3.

The images exhibited a better structural organization of OLEF_9, probably due to the presence of OLE powder in its polymeric matrix, compared to BF_9 (Figure 3a,c). BF_9 showed a different organization with several inhomogeneous areas due to the absence of an open porous structure, resulting in non-uniform crosslinking on the film’s surface (Figure 3a,b). Instead, porous features were revealed by OLEF_9, as shown in Figure 3d, helping the film matrix to absorb water and exudate the contents.

### 3.3. Film Expansion Profile

In order to recreate the wet wound surface environment and investigate the absorption of wound exudates into the film matrix, the 3D-printed samples were immersed into a gelatin solution and the hydration, expansion and degradation were evaluated.

**Figure 4:**
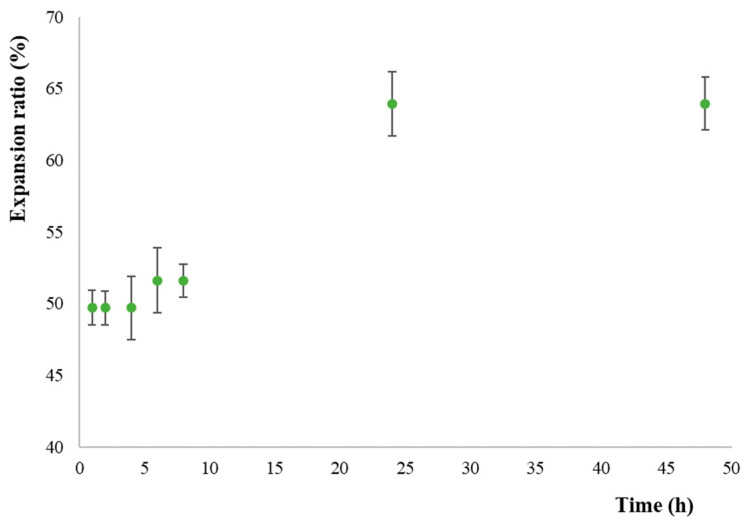
Expansion profile of OLEF_9: percentage increase in the expansion of the 3D-printed alginate/pectin-based film as a function of time.

The films expanded in all directions after slow hydration on the gelatin surface and the obtained data are shown in Figure 4.

Upon contact with a moist wound surface, a dry film absorbs wound exudates into its matrix. As a result, the film hydrates, expands, and eventually degrades and transforms into gels over the wound surface [48].

In our study, the expansion profile, evaluated using Equation (1), showed a significant increase in size after the time intervals of 1 and 6 h, reaching the peak of increase (63.94%) at 24 h. In addition, no degradation of the film was noted in the chosen time interval and OLEF_9 maintained its structure until 48 h.

### 3.4. Water Vapor Transmission Rate (WVTR)

A favorable microenvironment is required for wound healing, and one of the most significant features is an optimal moisture content. A low water vapor transmission rate could cause clinical problems such as a back pressure effect on the wound due to the build-up of exudate below the wound dressing. For this reason, tissue repair materials should have a WVTR in the range of 480–720 g·m^−2^·d^−1^.

In the present study, the WVTR of OLEF_9 was calculated to be 695.17 ± 14.18 g·m^−2^·d^−1^, which is an acceptable value since it falls in the range indicated by Cheng Y. and colleagues [49].

### 3.5. Total Phenolic Content and Antioxidant and Anti-Inflammatory Activities

The Folin–Ciocalteu assay was performed to quantify the Total Phenolic Content (TPC) and the amount of phenolic compounds was expressed as the catechin equivalents (CAE) in milligrams per g of sample (mg CAE/g) using a standard curve. The results showed that a good polyphenols content, equal to 13.15 ± 0.41 mg CAE/g, was found in the 3D-printed films due to the incorporated OLE powder.

In order to verify the total antioxidant activity, a phosphomolybdate assay was performed. Generally, at an acidic pH, antioxidant species reduce Mo(VI) to Mo(V), resulting in the formation of a green phosphate–Mo(V) complex. The high absorbance of OLEF_9 indicated significant antioxidant activity compared with the literature [50], being equal to 22.66 ± 0.52 mg CAE/g.

**Figure 5:**
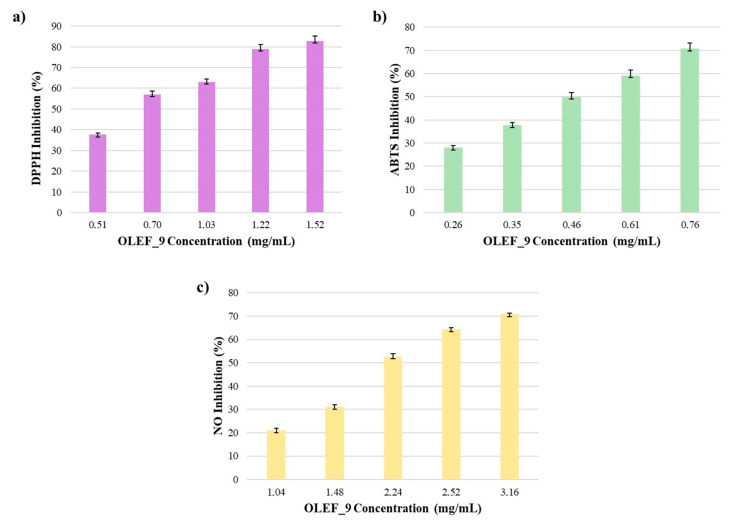
Antioxidant and anti-inflammatory properties of the 3D-printed alginate/pectin-based film incorporating OLE powder: (**a**) DPPH, (**b**) ABTS and (**c**) NO radical scavenging activities.

In order to evaluate the scavenging properties of OLEF_9, a lipophilic radical, such as DPPH, and a hydrophilic one, such as ABTS^•+^, were used. In both cases, the reaction depends on the ability of the antioxidant to donate hydrogen atoms. The DPPH radical is a stable radical that reduces to the yellow-colored compound diphenylpicrylhydrazine in the presence of antioxidants. At the same time, the ABTS^•+^ radical cation decolorization technique is based on the antioxidants that reduce ABTS^•+^ radicals. The mechanism of these reactions includes their ability to donate electrons and culminates in radical decolorization with a loss of color [45]. Therefore, the course of the reaction is directly related to the reducing capacity of the antioxidant. The ability of the antioxidant to act as a scavenger was measured in terms of IC_50_, which represents the sample concentration that is able to inhibit 50% of the radical; data were expressed as a percentage of the inhibition, as shown in Figure 5.

Under our conditions in the DPPH assay, OLEF_9 was shown to be a major radical scavenger, with an IC_50_ value of 0.66 ± 0.07 mg/mL; by applying higher quantities of sample (0.51–1.52 mg/mL), the percentage of radical inhibition increased (37.55–82.74%).

For the ABTS assay, the data shown in Figure 5b confirmed the efficiency of the 3D-printed film due to the presence of the olive leaf extract, which was more reactive with the hydrophilic radical. In this case, indeed, the assessed IC_50_ value was lower and equal to 0.47 ± 0.04 mg/mL.

Additionally, the anti-inflammatory efficacy was evaluated according to the reactivity of OLEF_9 toward Nitric Oxide (NO). NO is a proinflammatory mediator that, when combined with superoxide anions, produces the extremely toxic free radical peroxynitrite [51]. Based on these considerations, the items able to scavenge this kind of nitrogen radical present an anti-inflammatory behavior. The capacity of the prepared film to scavenge NO was examined and the resulting data, expressed as the radical inhibition percentage, are reported in Figure 5c. In detail, the developed patch showed an IC_50_ of 2.02 ± 0.14 mg/mL, which is indicative of good anti-inflammatory activity ascribable to the bioactive compounds contained in the olive leaf extract.

Data regarding the control patch BF_9 are not reported in Figure 5 because the examined blank film did not interfere in the performed assays and, thus, did not show any radical inhibition activities.

### 3.6. In Vitro Polyphenols Release Studies

The in vitro release studies were performed over a 48 h period following the dialysis membranes approach.

**Figure 6:**
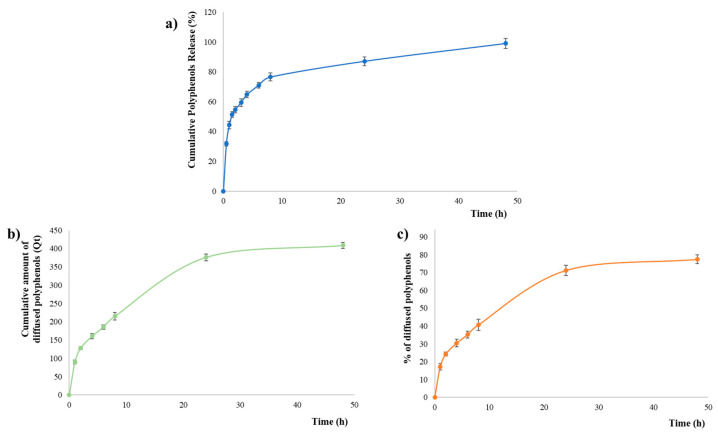
Evaluation of polyphenols release and diffusion from the prepared 3D-printed alginate/pectin-based film: (**a**) in vitro release profile according to the dialysis membranes method, (**b**) cumulative amount of diffused polyphenols per unit area (Q_t_) and (**c**) cumulative percentage of diffused polyphenols according to the Franz cells method.

The release media were evaluated via an indirect colorimetric method (Folin–Ciocalteu assay) for the quantification of polyphenols liberated from the prepared film and the assessed TPC was used as the total amount of polyphenols incorporated into OLEF_9. The in vitro cumulative release profile is illustrated in Figure 6a and, as it is possible to observe, the amount of released polyphenols was substantial.

In the first 2 h, a burst effect was noted, which may be due to the rapid dissolution of the surface polyphenols, and the amount of active molecules released from OLEF_9 was 54.55%. After 120 min, sustained release was also observed, amounting to 76.55% at the end of 8 h; the cumulative release of polyphenols increased progressively over time, reaching 100% after 48 h.

In order understand the mechanism of the release process, different kinetic models, such as zero-order, first-order, Higuchi, Hixson–Crowell and Korsmeyer–Peppas models, were applied using the following (Equations (6)–(10), respectively):

(6) $$\frac{M_{t}}{M_{\infty }}=K_{0}t$$

(7) $$\ln\left(1-\frac{M_{t}}{M_{\infty }}\right)=-K_{1}t$$

(8) $$\frac{M_{t}}{M_{\infty }}=K_{H}t^{1/2}$$

(9) $$M_{\infty }^{1/3}-M_{t}^{1/3}=Kt$$

(10) $$\frac{M_{t}}{M_{\infty }}=K_{P}t^{n}$$

where M_t_ represents the released amount of polyphenols at time t, M_∞_ is the total amount of polyphenols incorporated into the film, and K_0_, K_1_, K_H_, K and K_P_ are the zero-order, first-order, Hixson–Crowell, Higuchi and Korsmeyer–Peppas constants, respectively.

Based on the obtained coefficient of determination (R^2^) values (Table 2), the best fitting kinetic release model was chosen.

The in vitro release of polyphenols from OLEF_9 was best explained by the first-order kinetic model, which can be used to describe the release of active molecules from polymeric films and indicates a concentration-dependent mechanism in which a higher initial concentration suggests a more rapid release, as visible in the first 2 h (Figure 6a). According to this model, the kinetic release rate is affected by how the concentration of the therapeutic agent changes over time, and this is in line with the observed release profile.

In addition, when the obtained data were fitted using the Korsmeyer–Peppas equation, which has an R^2^ equal to 0.9631, the release exponent (*n*) was 0.3514. This parameter is highly useful since it indicates the type of release mechanism involved. For polymeric delivery systems characterized by a slab geometry, if *n* ≤ 0.5, a Fickian diffusion is observed, and this suggests that the drug diffuses through the hydrogel matrix [52,53]. In the other cases, if *n* = 1.0, the release is referred to as Case-II transport caused by system swelling, while if 0.5 < *n* < 1.0, the mechanism indicates an anomalous transport corresponding to a superposition of both diffusion and polymer relaxation. Based on the observed n value for OLEF_9, polyphenols were released from the 3D-printed film according to the diffusion mechanism.

### 3.7. In Vitro Diffusion Studies

In order to conduct a pilot study of the permeation assay, the experiments were performed by employing Franz diffusion cells and Strat-M^®^ membranes as a synthetic model [54].

The results were expressed as the cumulative quantity Q_t_ per unit area (μg/cm^2^) in time of polyphenols diffused through the membrane. As shown in Figure 6b, OLEF_9 exhibited a sustained diffusion, observing a release equal to 215.23 µg/cm^2^ in the first 8 h and reaching 408.88 µg/cm^2^ after 48 h. The observed release profile is due to the hydrophilic nature of the extract contained in the film and, thus, to the diffusion of polyphenols from polymeric film through the synthetic membrane [47].

In addition, the steady-state flux (J) and the permeability coefficient (K_p_) are other important parameters, where steady-state flux is the quantity of permeant crossing the membrane at a constant rate, while the permeability coefficient is calculated from the flux and the initial concentration (C_i_) of polyphenols in the donor compartment, as reported in Equation (11) [34]:

(11) $$K_{p}=\frac{J}{C_{i}}$$

To calculate the steady-state flux, the lag time, in which polyphenols penetrate the membrane and diffuse into the receptor fluid before reaching a steady state of diffusion, needs to be noticed. In detail, J was determined from the linear slope of the cumulative amount of diffused polyphenols vs. time curve, and its value was 11.632 ± 0.016 µg/cm^2^ h; meanwhile, the K_p_ was calculated according to Equation (10) and was equal to 23.87 × 10^−^^3^ ± 0.13 cm/h. This proves that polyphenols were released from the polymeric matrix and were able to constantly diffuse through the synthetic membrane.

### 3.8. Proliferative Effects of OLEF_9 and BF_9 on BJ Fibroblast Cells

The MTT assay was performed in order to evaluate the proliferative effects of OLEF_9 and BF_9 on human BJ fibroblast cells.

**Figure 7:**
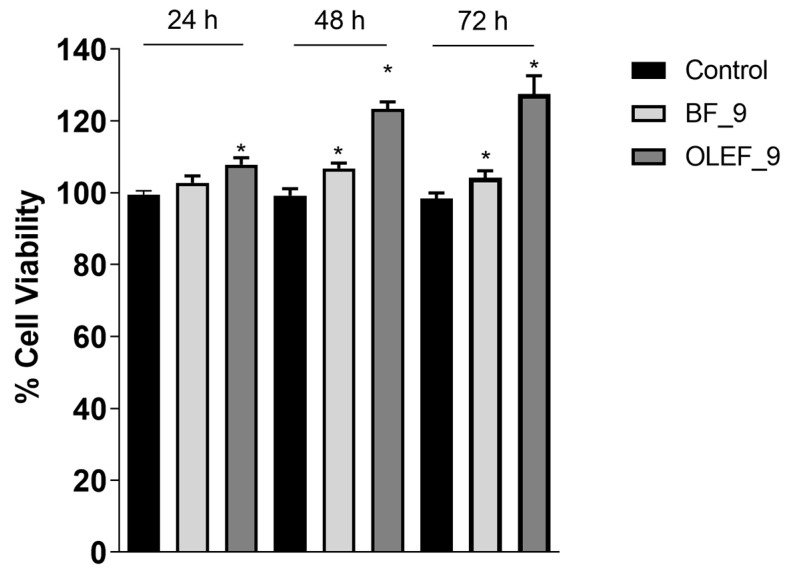
MTT cell proliferation assay. BJ cells treated with OLEF_9 and BF_9 for 24, 48, 72 h. Results are expressed as a percentage of the mean absorbance values, compared to the control, and represent the mean ± SE of three different experiments. * *p* < 0.01; compared to the control.

The experimental procedure relies on the capacity of viable cells to assimilate MTT. The test was performed at three experimental times: 24, 48 and 72 h. The results in Figure 7 show that OLEF_9 is able to increase cell proliferation, compared to both the negative control and the blank patch BF_9, in a time-dependent manner and that the greatest effect is obtained after 72 h of exposure.

### 3.9. Effects of OLEF_9 and BF_9 on Cell Motility, Collagen Expression and Collagen Levels in Fibroblast Cells

In order to demonstrate the ability of OLEF_9 to promote cell motility and, thus, its ability to heal wounds, a wound healing assay was performed on human BJ fibroblast cells.

The BJ fibroblast cells were starved in serum-free medium for 12 h. Upon reaching 100% confluence, a scratch was induced and cells were treated with OLEF_9 and BF_9. The scratch closure was monitored for 24 h using a microscope.

**Figure 8:**
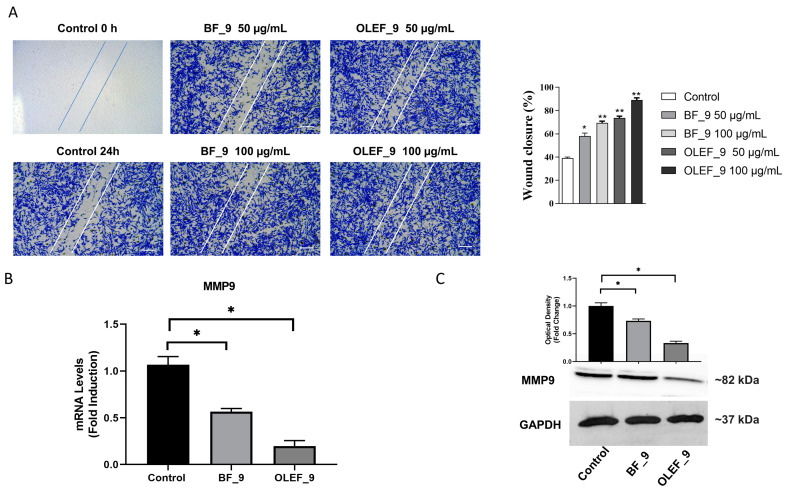
(**A**) Effects of OLEF_9 and BF_9 on cell motility. The wound healing scratch assay in cells treated with OLEF_9 and BF_9. After 24 h of treatment, the cells were stained with Brilliant Blue Coomassie and then photographed under an OLYMPUS BX-51 microscope at 10× magnification. The histogram represents the relative percentage of cut closure, calculated by image analysis using ImageJ software. Scale bar: 100 μm. (**B**,**C**) Real-time RT-PCR and immunoblotting assay of MMP-9 in BJ cells. GAPDH was used as a loading control. The histograms represent the mean average ± SD of three separate experiments in which the band intensities were evaluated in terms of the optical density arbitrary unit and expressed as the fold change over vehicle (−) for the OLEF_9 treatment * *p* < 0.05; ** *p* < 0.001.

The results showed that both the samples were able to increase wound healing compared to the negative control (Figure 8A).

Alginate, indeed, is characterized by several interesting properties, including anti-inflammatory, antibacterial and proliferative effects, which exert a main role in the healing process; in the literature, several studies report on the ability of alginate-based dressings to promote fibroblast proliferation and migration and collagen synthesis [55,56,57,58]. For example, Xi et al. described the preparation of a composite dressing, consisting of collagen, chitosan and alginate, that is able to induce fibroblast and endothelial cell migration [59]. Therefore, the ability of this polysaccharide to stimulate cell proliferation and motility could explain the results observed for the empty patch BF_9.

On the other hand, cells treated with the full patch, OLEF_9, showed more cell motility than cells treated with the BF_9 sample, and this is more evident at the concentration of 50 μg/mL. This behavior is ascribable to the presence of olive leaf extract, which consists of a mixture of active compounds including oleuropein and that acts as an accelerator of wound healing.

In line with the enhanced motility and invasiveness induced by OLEF_9 and BF_9 in BJ cells, we found a significant decrease in the mRNA and protein expression in MMP-9 in BJ cells after treatment with OLEF_9 and BF_9 (Figure 8B).

Fibroblasts are the main source of collagen synthesis in the dermis [60]. Torrecillas-Baena and collaborators reported complete healing at 14 days after the infliction of wounds on their murine experimental models, undoubtedly due to the increased accumulation of collagen in the extracellular matrix with EHO-85 treatment on fibroblasts and their activity [61]. This is supported by previous findings from the same research group, demonstrating that OLE protects dermal fibroblasts in vitro against the oxidative stress induced by hydrogen peroxide [62]. Moreover, other studies have shown OLE’s ability to act as a photoprotective, anti-inflammatory and antioxidant agent in dermal fibroblasts exposed to ultraviolet irradiation [63]. Additionally, some studies suggest that oleuropein, which is the most abundant polyphenol in OLE, has positive effects on fibroblasts. Thus, collagen-enriched nanovesicles containing oleuropein promote fibroblast proliferation and migration, accelerating skin wound healing [64]. Indeed, in incisional wounds in Bagg albino c (BALB/c) mice, the intradermal injection of oleuropein accelerated skin healing after seven days. Histological analyses of wounds in that study showed that oleuropein-treated wounds had lower inflammatory cell infiltration, a higher collagen content, and better re-epithelialization [15]. These data align with our preliminary findings, even though the application method differs.

In order to better understand the mechanisms responsible for the motility effects mediated by OLEF_9 and BF_9 in BJ fibroblast cells, the ability of the two tested samples to modulate the expression of Collagen I was evaluated.

**Figure 9:**
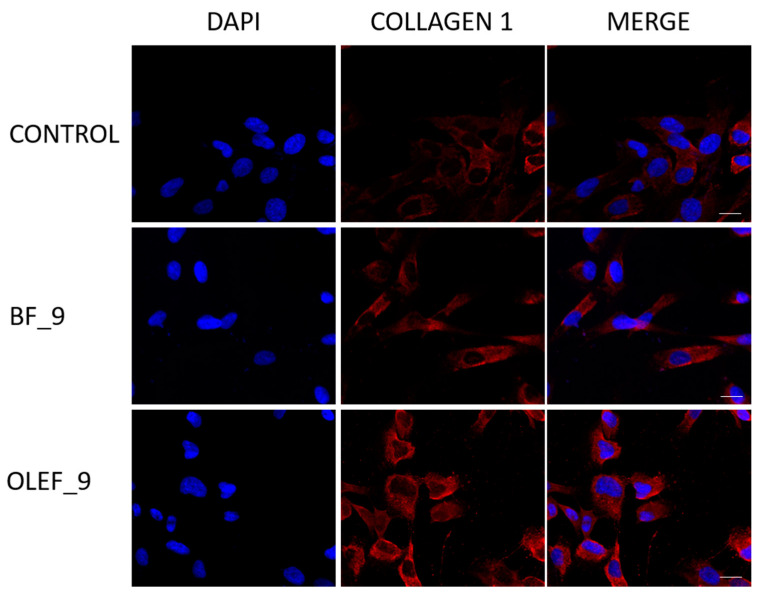
OLEF_9 and BF_9 treatment enhanced Collagen I expression in BJ cells. Collagen I. Expression was determined via immunofluorescence analysis. DAPI staining was used to visualize the cell nucleus. Scale bar: 25 μm.

The obtained data exhibited an increase in the expression levels of protein in the adopted models after treatment with OLEF_9 and BF_9 (Figure 9).

The mechanisms underlying the pathogenesis of skin ulcers in diabetics are various and still not completely understood. Underlying the skin lesions is the destruction of the epidermal structural barrier, caused by the reduced differentiation of the epidermis due to long-term hyperglycemia, and a reduction in the mechanical barrier of the dermis caused by alterations in collagen [65,66,67].

Several studies have demonstrated that extracellular matrix (ECM) hardening and reduced wound healing are based on collagen alteration, both structural (hyperglycemia-induced protein glycosylation) and in the synthesis and degradation process [68].

Collagen degradation is a process regulated by the balance between proteolytic enzymes of the metalloproteinase family (MMPs) and the inhibitors of tissue metalloproteinases (TIMPs). In diabetic patients, it has been observed that the pro-inflammatory and fibrotic factors (pro-inflammatory cytokines, ROS) released by the cells under hyperglycemic conditions induce the expression of matrix metalloproteinases, shifting the MMPs/TIMPs balance in favor of the prior and, thus, in support of ECM and growth factor degradation [69].

Furthermore, during the diabetic wound process, MMPs are involved in angiogenesis, the modulation of growth factor activity and the pathways related to inflammatory response, oxidative stress and apoptosis during wound healing [70,71,72,73,74].

Thus, targeting MMPs can be considered a new and promising strategy in diabetic ulcers.

The results obtained from our study show that OLEF_9, by inhibiting the expression of MMP9, metalloproteases of the gelatinase class, is able to decrease collagen degradation and increase the wound healing capacity in human fibroblast cells. Therefore, it should be interesting to further investigate the potential use of the prepared film in the treatment of diabetic ulcers.

### 3.10. Safety Assessment

#### 3.10.1. Cytotoxicity Evaluation by NRU

The cytotoxicity relative to OLEF_9 and BF_9 was assessed using the Neutral Red Uptake (NRU) test on BALB/3T3 murine fibroblast cells. The NRU test was performed according to ISO 10993-5:2009 [37].

**Figure 10:**
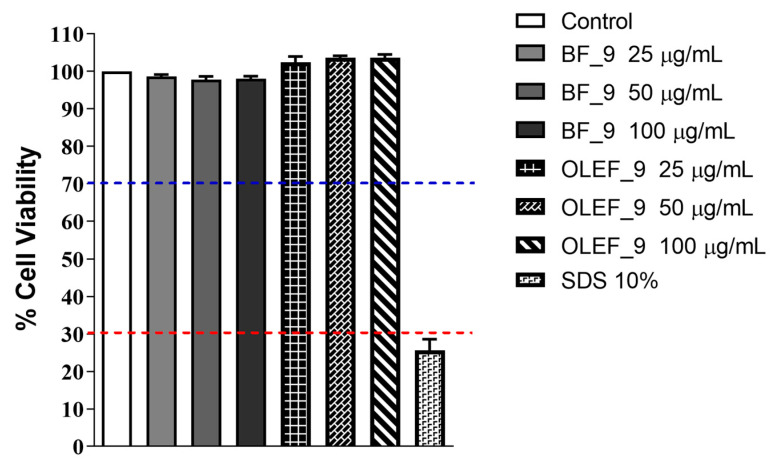
Clone A31 Balb/3T3 fibroblast cell viability NRU test (%) in the absence or in the presence of increasing doses of OLEF_9 and BF_9. Each column represents the mean ± SD of three wells/group. RED LINE: strongly cytotoxic, cell viability <30%, BLUE LINE: non cytotoxic, cell viability <70%.

The results, reported in Figure 10, show that the viability of cells treated with OLEF_9 and BF_9 at increasing concentrations does not decrease compared to the negative control (untreated cells). From the obtained results, it can be shown that OLEF_9 and BF_9 are not cytotoxic.

Prior to performing the NRU, the integrity of the used cell culture was assessed via microscopic observation after 24 h of incubation. The biological reactivity (abnormalities and cellular degeneration) was classified by assigning a grade from 0 to 4 according to ISO 10993-5:2009 [37].

The BALB/3T3 fibroblast cells, used as an experimental model, showed normal morphology and did not present any biological reactivity after incubation with the vehicle medium and the tested items, such as BF_9 and OLEF_9. On the contrary, the sample treated with the positive control (SDS 10%) had a reactivity value of 4.

Therefore, the obtained results confirmed the non-toxicity of the examined samples.

#### 3.10.2. Determination of In Vitro Skin Sensitization

In order to assess the sensitizing potential of OLEF_9 and BF_9, the Human Cell Activation Test (hCLAT) was performed according to OECD 442E.

This test is used to measure the CD86 and CD54 expression levels in THP-1 cells. The expression levels of the markers are measured according to their activation capacity via flow cytometry after 24 h of exposure to eight serial concentrations of the substance. The concentrations tested are chosen from a preliminary cytotoxicity study that determines the CV75 of the compound on cells.

In order to investigate the sensitization of the studied items, CD86 and CD54 RFI% values were utilized. A positive prediction of sensitization is established when the RFI% value for CD86 is ≥150% and/or when the RFI% value for CD54 is ≥200% in a minimum of two independent runs. On the contrary, a negative prediction of sensitization is assumed if the RFI% value for CD86 is <150% and/or if the RFI% value for CD54 is <200%.

The results provided in Table 3 confirm that the tested samples were not skin sensitizers.

#### 3.10.3. Hemolytic Effects of OLEF_9 and BF_9 on Peripheral Blood

In order to test the toxicity of the OLEF_9 and BF_9 samples in blood, the hemolysis test was carried out. Peripheral blood was collected from a healthy adult; the test was performed by treating the red blood cells (RBCs) with increasing doses of OLEF_9 and BF_9.

**Figure 11:**
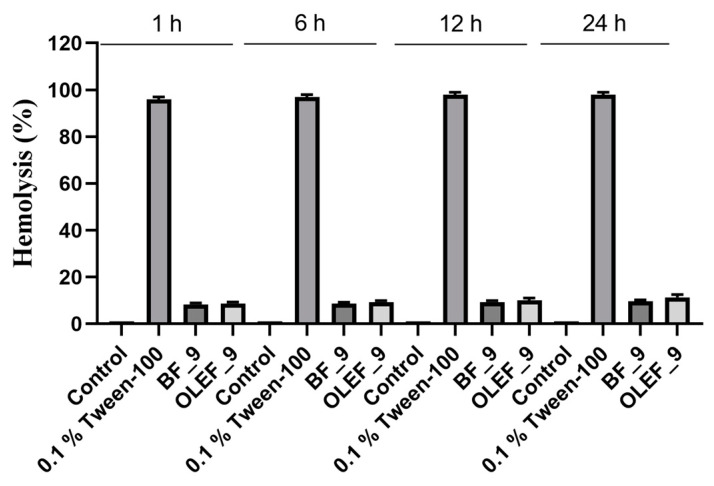
Hemolytic effects of OLEF_9 and BF_9: RBCs treated with PBS (Control), 0.1% Tween-100, OLEF_9 and BF_9 (100 µg mL^−1^) for 1, 6 or 24 h. Histograms represent the relative percentage of hemolysis from three different experiments, each performed with triplicate samples. *p* values were calculated against 0.1% Tween-100.

Figure 11 reveals the non-hemolytic behavior of the patches, with an acceptable rate of hemolysis even at the highest tested concentration (100 µg mL^−^^1^).

## 4. Conclusions

The focus of this study is on the development of a 3D-printed patch, based on alginate and pectin, for the treatment of wounds using bioactives derived from natural waste products, such as olive leaves. The inclusion of the olive leaf extract imparted the film with a rich reservoir of polyphenols, conferring antioxidant and anti-inflammatory properties, which are desirable in wound care.

The release profiles revealed an almost complete release of the polyphenols from the 3D-printed patch within 48 h. Moreover, the prepared polymeric film demonstrated the ability to promote cell motility and wound healing and to increase collagen I expression in BJ fibroblast cells. In order to verify the structural properties of OLEF_9, WVTR (Water Vapor Transmission Rate) and expansion studies were performed, revealing the patch’s capacity to absorb exudates and maintain an optimal moisture balance. Finally, biocompatibility studies were conducted and the performed NRU, hemolysis and h-CLAT assays confirmed the material’s safety.

The obtained results suggested the effectiveness and potential use of OLEF_9 in wound healing applications, opening perspectives for its use in the biomedical and therapeutic fields. However, additional studies are needed to more comprehensively assess the efficacy of the developed 3D-printed patch in an in vivo model due to the complexity of the healing process.

## Figures and Tables

**Table 1:** Composition of the 3D-printable INKs and viscosity analyses.

3D-Printable INKs	OLE Powder (% *w*/*v*)	ALG (% *w*/*v*)	PEC (% *w*/*v*)	GLY (% *w*/*v*)	Viscosity (Pa·s)
INK_1	1.0	1.5	1.5	1.5	4.1 ± 0.2
INK_2	1.0	1.5	1.5	3.0	7.6 ± 0.2
INK_3	1.0	2.0	2.0	5.0	13.0 ± 0.4

**Table 2:** Release kinetic models.

Sample	Zero-Order	First-Order	Higuchi	Hixson–Crowell	Korsmeyer–Peppas	
	R^2^	R^2^	R^2^	R^2^	R^2^	N
*OLEF_9*	0.7012	0.9692	0.8658	0.9360	0.9631	0.3514

**Table 3:** RFI% value of CD54 and CD86 on THP-1 monocytes.

Samples	CD54 *	CD86 *
BF_9	58.21	70.19
OLEF_9	62.71	72.31
Negative control	49.24	61.48
Positive control (NISO_4_)	237.51	181.231

* The sample is a skin sensitizer where CD86 > 150 and CD54 > 200.

## Data Availability

The original contributions presented in the study are included in the article/supplementary material, further inquiries can be directed to the corresponding author/s.

## Supplementary Materials

The following supporting information can be downloaded at: https://www.mdpi.com/article/10.3390/pharmaceutics16010099/s1, Table S1: CaCl_2_ concentration and time of crosslinking reaction.
