# Supplementary material for: 3D-Printed Alginate/Pectin-Based Patches Loaded with Olive Leaf Extracts for Wound Healing Applications: Development, Characterization and In Vitro Evaluation of Biological Properties
Source: Pharmaceutics. 2024 Jan 11;16(1):99. doi: 10.3390/pharmaceutics16010099 (PMC10819698; doi:10.3390/pharmaceutics16010099)
Supplement: Supplementary file 1 [file pharmaceutics-16-00099-s001.zip › pharmaceutics-2744614-supplementary.pdf]

Table S1. $CaCl_2$ concentration and time of crosslinking reaction.

**SAMPLE**	**$CaCl_2$ (mM)**	**Crosslinking time (sec)**
OLEF_1	90.11	20
OLEF_2	90.11	40
OLEF_3	90.11	60
OLEF_4	135.16	20
OLEF_5	135.16	40
OLEF_6	135.16	60
OLEF_7	180.21	20
OLEF_8	180.21	40
OLEF_9	180.21	60
